# MLP-deficient human pluripotent stem cell derived cardiomyocytes develop hypertrophic cardiomyopathy and heart failure phenotypes due to abnormal calcium handling

**DOI:** 10.1038/s41419-019-1826-4

**Published:** 2019-08-13

**Authors:** Xiaowei Li, Wen-Jing Lu, Ya’nan Li, Fujian Wu, Rui Bai, Shuhong Ma, Tao Dong, Hongjia Zhang, Andrew S. Lee, Yongming Wang, Feng Lan

**Affiliations:** 1 Beijing Laboratory for Cardiovascular Precision Medicine, The Key Laboratory of Remodeling‐Related Cardiovascular Disease, The Key Laboratory of Biomedical Engineering for Cardiovascular Disease Research, Ministry of Education, Beijing Collaborative Innovation Center for Cardiovascular Disorders, Anzhen Hospital, Capital Medical University, 100029 Beijing, China; 2 Beijing Institute of Heart, Lung and Blood Vessel Diseases, 100029 Beijing, China; 3 Center for Clinical Translation and Innovation, Peking University Shenzhen Graduate School, Shenzhen, 518055 China; 4 Shenzhen Bay Laboratory, Shenzhen, 518055 China; 5 The State Key Laboratory of Genetic Engineering and MOE Key Laboratory of Contemporary Anthropology, School of Life Sciences, Fudan University, Shanghai, 200438 China

**Keywords:** Mechanisms of disease, Embryonic stem cells, Stem-cell differentiation

## Abstract

Muscle LIM protein (MLP, *CSRP3*) is a key regulator of striated muscle function, and its mutations can lead to both hypertrophic cardiomyopathy (HCM) and dilated cardiomyopathy (DCM) in patients. However, due to lack of human models, mechanisms underlining the pathogenesis of MLP defects remain unclear. In this study, we generated a knockout MLP/CSRP3 human embryonic stem cell (hESC) H9 cell line using CRISPR/Cas9 mediated gene disruption. *CSRP3* disruption had no impact on the cardiac differentiation of H9 cells and led to confirmed MLP deficiency in hESC-derived cardiomyocytes (ESC-CMs). MLP-deficient hESC-CMs were found to develop phenotypic features of HCM early after differentiation, such as enlarged cell size, multinucleation, and disorganized sarcomeric ultrastructure. Cellular phenotypes of MLP-deficient hESC-CMs subsequently progressed to mimic heart failure (HF) by 30 days post differentiation, including exhibiting mitochondrial damage, increased ROS generation, and impaired Ca^2+^ handling. Pharmaceutical treatment with beta agonist, such as isoproterenol, was found to accelerate the manifestation of HCM and HF, consistent with transgenic animal models of MLP deficiency. Furthermore, restoration of Ca^2+^ homeostasis by verapamil prevented the development of HCM and HF phenotypes, suggesting that elevated intracellular Ca^2+^ concentration is a central mechanism for pathogenesis of MLP deficiency. In summary, MLP-deficient hESC-CMs recapitulate the pathogenesis of HCM and its progression toward HF, providing an important human model for investigation of CSRP3/MLP-associated disease pathogenesis. More importantly, correction of the autonomous dysfunction of Ca^2+^ handling was found to be an effective method for treating the in vitro development of cardiomyopathy disease phenotype.

## Introduction

Muscle LIM protein (MLP), encoded by *CSRP3*, is primarily expressed in cardiomyocytes and skeletal muscle cells and consists of 194 amino acids with two LIM domains^1^. These LIM domains mediate interactions with a variety of proteins in different subcellular regions, including the cytoplasm and the nucleus. In the cytoplasm of cardiomyocytes, MLP has been found in the sarcomeres, intercalated disks, and costameres of striated myocytes. In addition, MLP can directly affect F-actin cytoskeleton dynamics by enhancing CFL2-dependent F-actin depolymerization^2^ and stabilizing actin filaments into bundles^3^. These studies suggest that MLP plays an integral role in sarcomeric structure. Previous studies have shown that mutations (missense, small insertions, and deletions) in *CSRP3* are associated with hypertrophic cardiomyopathy (HCM)^4,5^ and dilated cardiomyopathy (DCM)^6^ in humans. In addition, MLP has been shown to be significantly downregulated in chronic human heart failure (HF)^7^. In mouse models, MLP-deficient mice develop dilated DCM with hypertrophy after birth, and are the first transgenic animal model of HF^1^. However, the underlying molecular mechanisms of *CSRP3* mutation-induced cardiomyopathy are not well understood.

Despite the existence of genetically modified mouse models for MLP deficiency^1,8^, phenotypic results have shown significant discrepancies between identical CSRP3 mutations in animals and humans. For example, MLP^W4R/+^ transgenic mice are observed to develop features of HCM and HF^8^, whereas MLP^W4R/+^ is a known to be a benign polymorphism in humans carrying the mutation^4,9^. Efforts to obtain primary human cardiac tissue to resolve these discrepancies have not been successful due to difficulties in obtaining and culturing human cardiac tissue in vitro. Advances in disease modeling using human pluripotent stem cells (PSCs) in recent years have provided a novel pathophysiological approach to study the mechanics of disease and discover new targets for pharmacological intervention^10^.

We thus created a MLP-deficient cardiomyocyte model using from *CSRP3*^−/−^ hESC-H9 cells, to elucidate the mechanism of cardiomyopathy and HF caused by MLP defects. We report here that MLP-deficient human embryonic stem cell-derived cardiomyocytes (hESC-CMs) recapitulate the phenotypes of HCM and HF. Mechanistically, impaired calcium handling is a major mechanism underlying the pathogenesis of MLP-associated HCM and HF, which can be prevented by pharmaceutical blockade of calcium entry into the cell.

## Results

### Generation of homozygous *CSRP3*^−/−^ hESCs

**Fig. 1:**
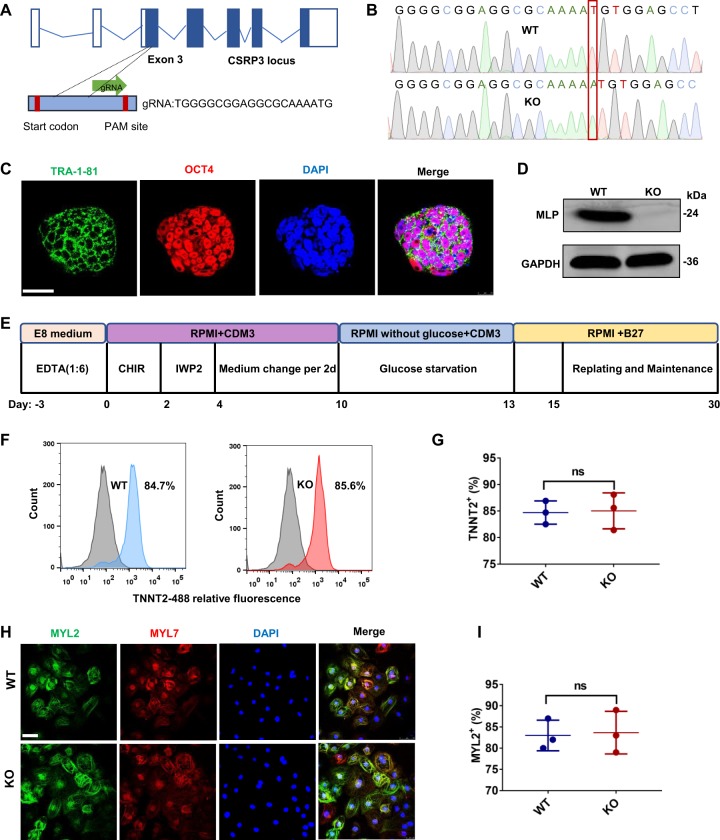
*CSRP3* knockout does not affect pluripotency of hESCs and differentiation into hESC-CMs. **a** Structure of the CSRP3 locus showing localization of gRNA for epiCRISPR/Cas9 editing. **b** Sequence chromatograms demonstrate one base insertion in *CSRP3* knockout (MLP KO) colonies. **c** Immunofluorescence staining of MLP KO colonies for the pluripotency markers TRA-1-81 and OCT4. Scale bar, 50 μm. **d** Immunoblot analysis of MLP in WT and MLP KO CMs at day 15. **e** Protocol for cardiac differentiation using small molecule-based methods. **f**, **g** Flow cytometry analysis for TNNT2 from representative WT and MLP KO differentiation protocols prior to purification at day 10. **h** Immunostaining for expression of MYL2 (green) and MYL7 (red) in WT and MLP KO CMS at day 30. **i** Quantification of ventricular marker MYL2 in WT (*n* = 382) and MLP KO CMS (*n* = 403). Scale bar, 50 μm. Results are presented as means ± S.E.M. of three independent experiments. ns, not significant, unpaired two-sided Student’s *t* test

To generate the human PSC model of MLP deficiency, we designed a single-guide RNA targeting exon 3 of CSRP3 using the epiCRISPR/Cas9 gene editing system (Fig. 1a). H9 hESCs were then electroporated with a plasmid containing sgRNA and epiCRISPR/Cas9^11^. After selection with puromycin, the resistant colonies were expanded for PCR screening verification. Among 12 clones, we found one homozygous *CSRP3* knockout line with 1-nucleotide insertion resulting in a frame-shifted coding sequence with premature stop codon at 14th amino acid, resulting in homozygous null alleles (Fig. 1b). *CSRP3*^−/−^ hESC colonies exhibited normal morphology (Fig. S1A). We further confirmed that *CSRP3*^−/−^ lines expressed the human pluripotency markers TRA-1-81 and OCT4 (Fig. 1c) and had normal karyotype (Fig. S1B). Finally, CSRP3 knockout was not observed to affect the pluripotency of hESC evidenced by teratoma formation in vivo (Fig. S1C) and differentiation into all three embryonic germ layers (Fig. S1D). Moreover, we predicted the top ten off-target sites for sgRNA using an online analysis software (crispr.mit.edu) and found no off-target mutations in *CSRP3*^−/−^ hESCs by DNA sequencing (data not shown)^12^.

### MLP deficiency caused by CSRP3^−/−^ does not affect cardiac differentiation of hESCs

Since MLP is primarily expressed in cardiomyocytes, we differentiated CSRP3^−/−^ lines (MLP KO) and the parental H9 cells (WT) toward cardiomyocyte lineages (hESC-CMs) using small molecule-based protocols (Fig. 1e)^13^. At day 15 post differentiation, we validated the effect of CSRP3 knockout by western blot and observed absence of MLP protein in MLP KO hESC-CMs (Fig. 1d). Since MLP can serve as a positive regulator of myogenesis and promotes myogenic differentiation at the nucleus^14^, we next evaluated the impact of MLP deficiency on cardiac differentiation efficiency. Flow cytometry staining for cardiac Troponin T (cTnT) showed both the WT and MLP KO derived cells contained close to 85% positive cTnT cells by flow cytometry at day 10 post differentiation (Fig. 1f, g) and ~98% positive cTnT cells after purification at day 15 post differentiation (Fig. S1E, F). There was no statistical difference between the groups (*P* = 0.43). Double immunostaining for MYL2 and MYL7 showed both 83% of WT and MLP KO hESC-CMs were positive for the ventricular marker MYL2^+^ (Fig. 1h, i). These findings suggested that MLP deficiency has no significant effect on myocardial differentiation.

### MLP-deficient hESC-CMs recapitulate HCM phenotypes in vitro

**Fig. 2:**
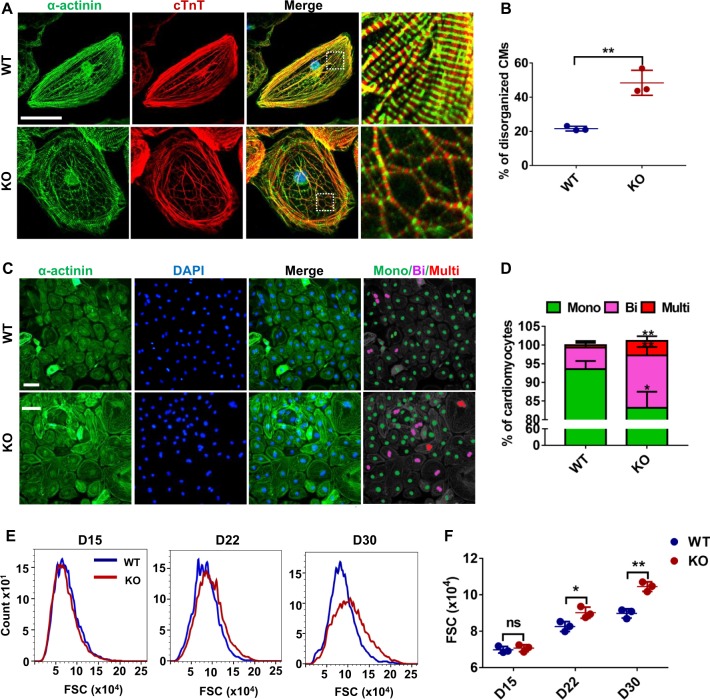
Phenotyping hypertrophic cardiomyopathy in MLP-deficient hESC-CMs. **a** Immunostaining of sarcomeric α-actinin (green) and cTnT (red) demonstrates sarcomeric disarray in MLP KO CMs at day 30. Scale bar, 50 μm. **b** Compared with WT hESC-CMs (*n* = 189), a significant higher percentage of MLP KO hESC-CMS (*n* = 191) showed disorganized sarcomeric α-actinin staining pattern in greater than one fourth of the total cellular area. **c** Images of α-actinin/DAPI-immunostained hESC-CMs and **d** quantification of mono-, bi-, and multi-nucleation in WT (*n* = 267) and MLP KO (*n* = 259) hESC-CMs. Scale bar, 50 μm. **e**, **f** Calibration of forward scatter (FSC; 10,000 cells/sample, *n* = 3) showing an increased cellular size beginning 22 days post cardiac differentiation in MLP KO CMS. **f** Results are presented as means ± S.E.M. of three independent experiments. *P < 0.05; **P < 0.01; ns, not significant, unpaired two-sided Student’s *t* test

Since myofibrillar disarray is found in HCM patient specific hiPSC-CMs^15^ and have been shown to result in cardiomyocyte dysfunction^16^, we next examined the morphological changes of MLP-deficient CMs. Compared with WT hESC-CMs at day 30 post differentiation, immunostaining of cTnT and α-actinin of MLP-deficient hESC-CMs showed a significant higher frequency of sarcomeric disorganization, which was defined as disorganized sarcomeric α-actinin staining pattern observed in over 25% of the total cellular area with reference to previous studies^17^ (Figs. 2a, b and S2A). Multinucleation^18^ and increased cardiomyocyte size^19^ are also critical features of HCM in human cardiomyocytes^20^. We thus used a high-throughput (10,000 cells/sample) and nonsubjective flow cytometry method to measure hESC cardiomyocytes size via flow cytometry measurement of forward scatter using calibration spheres for cell size determination^21^. As previously reported^22^, only a small number of (<7%) WT hESC-CMs were observed to be binuclear or multinuclear, while MLP KO hESC-CMs exhibited significantly higher percentages of bi- or multi-nucleation (about 17%) at day 30 post differentiation (Fig. 2c, d). In addition, compared with WT hESC-CMs, MLP KO hESC-CMs gradually exhibited cell enlargement from day 22 post differentiation onward (Figs. 2e, f and S2B). These results suggest that MLP deficiency leads to cytoarchitectural features of HCM in hPSC-CMs.

### MLP deficiency leads to impaired calcium handling

**Fig. 3:**
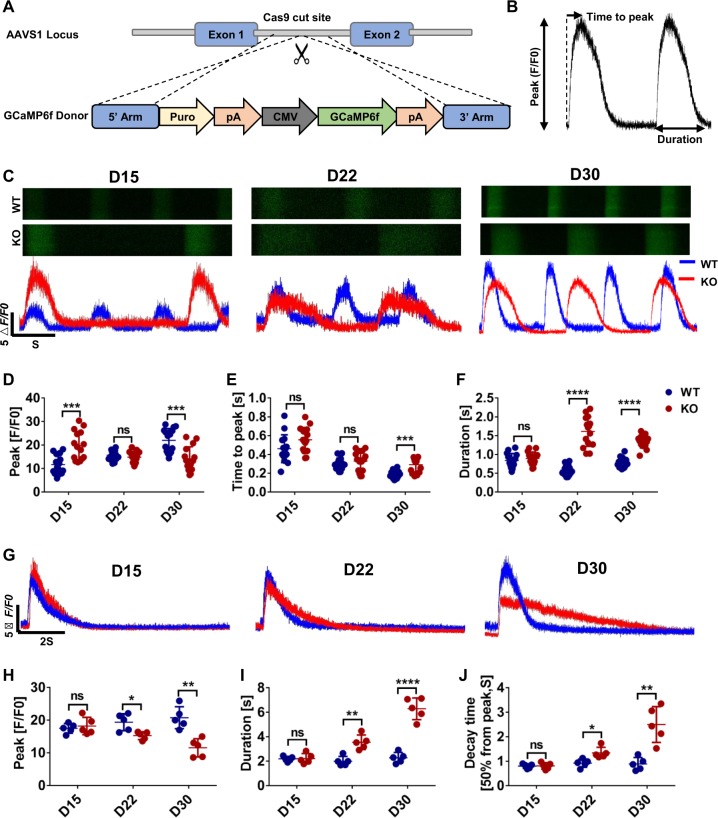
MLP-deficient hESC-CMs exhibit abnormal Ca^2+^ handling properties. **a** Schematic demonstrating the GCaMP-expression cassette that was integrated into AAVS1 of WT and MLP KO hESCs via nickase CRISPR/Cas9 editing. **b** Space-averaged calcium transients showing parameters measured for analysis of calcium handling. **c** Representative line-scan images in WT-GCaMP and MLP KO-GCaMP hESC-CMs at days 15, 22, and 30. **d**–**f** Quantification of peak, time to peak, and calcium transient duration in WT-GCaMP and MLP KO-GCaMP hESC-CMs (*n* = 15 cells per group). **g** Representative Ca^2+^ transients induced with 10 mM caffeine in Ca^2+^-free conditions between WT-GCaMP and MLP KO-GCaMP hESC-CMs at days 15, 22, and 30 (*n* = 5 cells per group). **h**–**j** Peak amplitude, transient duration, and decay time in caffeine-evoked Ca^2+^ transients WT-GCaMP and MLP KO-GCaMP hESC-CMs. Results are presented as means ± S.E.M. of three independent experiments. *P < 0.05; **P < 0.01; ***P < 0.001; ****P < 0.0001; ns, not significant, unpaired two-sided Student’s *t* test

Calcium handling plays a fundamental role in regulation of excitation–contraction in both skeletal and cardiac myocytes, dysfunction of which is commonly involved in the development of HCM and HF^10,23^. To examine the impact of MLP deficiency on calcium handling, we applied nickase Cas9 to introduce the green fluorescent calcium-modulated protein 6 fast type (GCaMP6f) calcium sensor into the AAVS1 locus of the WT and MLP KO H9 hESC lines as previously reported^24^ (Fig. 3a). Recording of calcium transients at days 15, 22, and 30 (Fig. 3b, c) show that compared with WT hESC-CMs, MLP KO hESC-CMs have slower beating rate (Figs. 3c and S3A). At day 15, the calcium release amplitude of MLP KO CMs was significantly higher than WT CMs, but gradually decreased (Fig. 3c, d). At day 30, compared with WT hESC-CMs, MLP KO hESC-CMs exhibited lower calcium release amplitude and longer time to peak (Fig. 3c–e). Beginning at day 22, MLP KO hESC-CMs started to demonstrate longer transient durations (Fig. 3c, f) and prolonged decay times (Fig. S3B) compared with WT hESC-CMs. Adjustment to heart rate variation did not impact prolonged transients of MLP KO hESC-CMs as compared with WT hESC-CMs (Fig. S3C). Interestingly, increased calcium release amplitude was observed to occur in MLP KO CMs before cellular hypertrophy (Figs. 2f and 3d), suggesting that abnormal Ca^2+^ handling may be a causal factor for the induction of HCM phenotype in MLP-deficient hESC-CMs.

To further profile calcium handling properties, we also measured caffeine-induced calcium release from the SR of WT and MLP KO hESC-CMs at days 15, 22, and 30 (Figs. 3g and S3D). Beginning at day 22, MLP KO hESC-CMs exhibited relatively smaller amplitudes, longer duration, and prolonged decay time compared with WT hESC-CMs (Figs. 3g, j and S3D), suggesting that MLP KO hESC-CMs have reduced SR calcium storage and impaired function of Ca^2+^ pumping and Ca^2+^ release channels in the SR and plasma membranes. Consistently, increased *CACNA1C* and *RYR2* expression (Fig. S3E, F) and decreased SERCA 2a levels were observed in MLP KO hESC-CMs by qPCR (Fig. S3G) and western blot (Fig. S3H, I). These results indicate that MLP deficiency decreases the rate of calcium uptake and release in hESC-CMs as demonstrated in other models of HCM^25,26^ and HF^27^.

### MLP-deficient cardiomyocytes exhibit elevated activity of hypertrophic signaling pathways

**Fig. 4:**
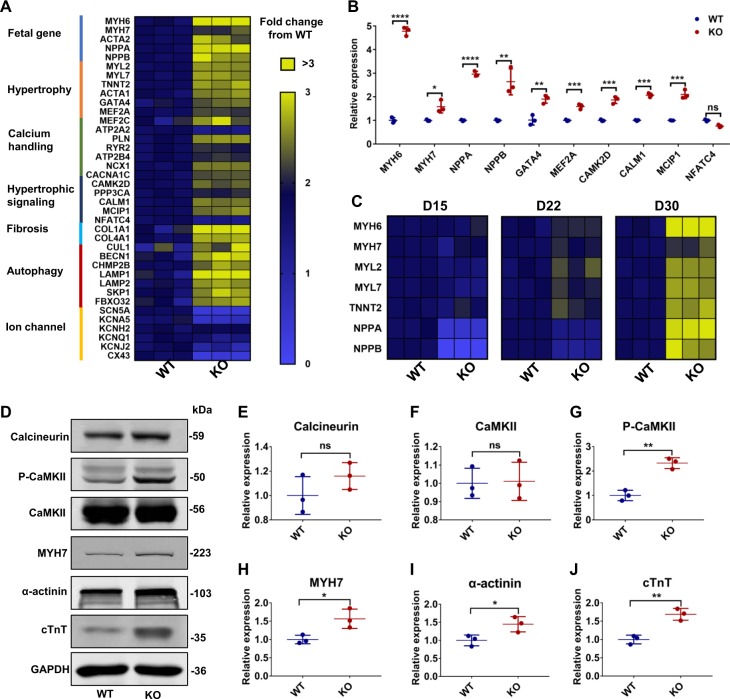
Increased gene expression of hypertrophic signaling pathways in MLP-deficient hESC-CMs. **a** Heatmap showing changes in the expression of genes involved in HCM signaling pathways, calcium handling, fibrosis, and autophagy in WT and MLP KO hESC-CMs at day 30. **b** qRT-PCR analysis of HCM related genes in WT and MLP KO CMs at day 30. **c** Heatmap showing the expression of HCM associated genes at days 15, 22, and 30 of cardiac differentiation. **d** Immunoblot analysis of HCM signaling (calcineurin A, CaMKIIδ, and phosphorylated CaMKII) and sarcomere proteins (MYH7, α-actinin, and cTnT) in WT and MLP KO hESC-CMs at day 30. **e**–**j** Quantification of calcineurin A, CaMKIIδ, P-CaMKII, MYH7, α-actinin, and cTnT normalized by GAPDH in WT and MLP KO hESC-CMs. Results are presented as means ± S.E.M. of three independent experiments. *P < 0.05; **P < 0.01; ***P < 0.001; ****P < 0.0001; ns, not significant, unpaired two-sided Student’s *t* test

To understand how MLP deficiency leads to cardiomyopathy, we next accessed the expression of a panel of cardiomyopathy related genes at day 30 by qRT-PCR. Compared with WT hESC-CMs, MLP KO hESC-CMs displayed increased expression of genes involved in fetal development, calcium handling, hypertrophic signaling pathways, and autophagy (Fig. 4a–c). From this transcriptional data, we found significantly elevated activation of hypertrophic signaling in MLP KO hESC-CMs. To further examine signaling pathways involved^28,29^, we performed western blot for Ca^2+^-calcineurin–NFAT and Ca^2+^-Calmodulin-CaMKII. As compared with WT hESC-CMs, MLP KO hESC-CMs exhibited higher levels of P-CaMKII, which is a major mediator of hypertrophic signaling (Fig. 4d, e, g). Furthermore, MLP KO hESC-CMs exhibited higher level of MYH7, cTnT, and α-actinin, consistent with increased sarcomeric gene expression, which is a known feature of HCM (Fig. 4d, h–j)^19^.

Because clinical symptoms of HCM usually take decades to manifest in affected individuals^30^, we also examined temporal gene expression in MLP KO hESC-CMs as compared with WT hESC-CMs by qRT-PCR. We found that from day 15 to day 30, the relative expression of hypertrophy related genes gradually increased in MLP KO hESC-CMs (Figs. 4c and S4A–G), and began to pick up in earnest around day 22 for genes such as *MYH6*, *MYH7*, *MYL2*, *MYL7*, and *TNNT2*, while *NPPA* and *NPPB* were significantly increased by day 30 (Figs. 4c and S4A–G).

### MLP-deficient CMs develop mitochondrial dysfunction associated with HF

**Fig. 5:**
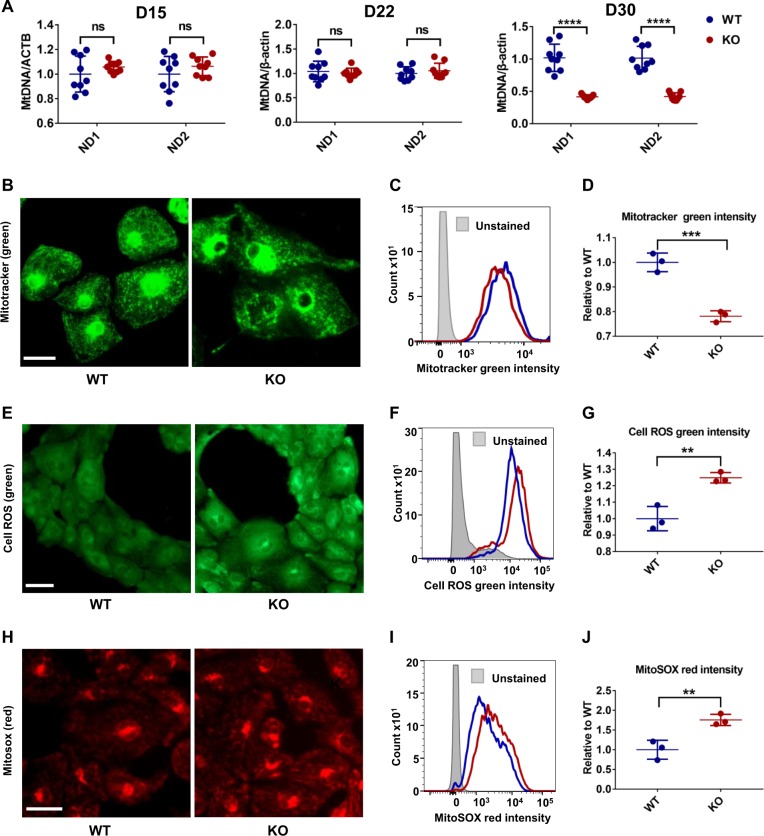
Mitochondrial damage and increased ROX in MLP-deficient CMs. **a** qPCR analysis of mitochondrial DNA (*ND1* and *ND2*) to nuclear DNA (β-actin) ratio at days 15, 22, and 30 of cardiac differentiation (*n* = 9). **b** Mitotracker images and **c**, **d** quantification of Mitotracker green intensity obtained by flow cytometry demonstrates a significantly reduced fluorescence intensity in MLP KO hESC-CMs at day 30 as compared with WT hESC-CMs (*n* = 3). Scale bar, 50 μm. **e**–**g** Cell ROS green intensity and **h**–**j** MitoSox Red intensity suggests increase in ROS for MLP KO hESC-CMs as compared with WT hESC-CMs (*n* = 3). Scale bar, 50 μm. Results are presented as means ± S.E.M. of three independent experiments. **P < 0.01; ***P < 0.001; ****P < 0.0001; ns, not significant, unpaired two-sided Student’s *t* test

Previous studies have shown that cardiomyopathies and HF are commonly associated with pathological events such as mitochondrial dysfunction, energy depletion, increased generation of ROS, and cardiac dysfunction^31,32^. MLP-deficient mice have also been shown to exhibit regional absence of mitochondria and energy depletion^33^. In addition, increased intracellular basal [Ca^2+^] caused by MLP deficiency has been shown to damage mitochondria^34^. Thus, we next quantified mitochondrial content in MLP KO and WT hESC-CMs by comparing the amount of the mitochondrial-specific *ND1* and *ND2* genes to the housekeeping *ACTB* (encoding β-actin) at days 15, 22, and 30^35^. Beginning at day 30 post differentiation, MLP KO hESC-CMs exhibited a significant decrease in mtDNA/nDNA ratio compared with WT hESC-CMs (Fig. 5a). To further investigate the impacts of MLP deficiency on the metabolism of hESC-CMs, we next assessed mitochondrial content, total cellular ROS, and mitochondrial-specific ROS by flow cytometry using Mitotracker, Cell ROS green, and MitoSOX, respectively, at day 30. Compared with WT hESC-CMs, the mitochondrial content of MLP KO CMs was significantly reduced (Fig. 5b–d). Moreover, the staining pattern of Mitotracker in MLP KO hESC-CMs demonstrated that many mitochondria were punctate and fragmented (Fig. S5A) as compared with normal linear morphology in WT hESC-CMs, indicating that MLP KO hESC-CMs had hallmarks of mitochondrial damage. Flow cytometry assessment of cell ROS (Fig. 5e–g) and MitoSOX (Fig. 5h–j) staining demonstrated that both total cellular and mitochondrial-specific ROS are significantly elevated in MLP KO hESC-CMs, which is consistent with features of mitochondrial injury^32^.

Since damaged mitochondria can lead to decreased ATP production and increased AMP/ATP ratio, we next determined the functional change of mitochondria by AMP-activated protein kinase (AMPK) activity, a “metabolic sensor”, that can be activated by ATP depletion and increased AMP/ATP ratio^36^. MLP KO hESC-CMs had a significantly higher P-AMPK levels compared with WT CMs at day 30 (Fig. S5B, C). These findings suggest that MLP deficiency may lead to HF phenotypes due to mitochondrial damage, increased ROS, and energy depletion.

### MLP-deficient cardiomyocytes exhibit early failure after isoproterenol treatments

**Fig. 6:**
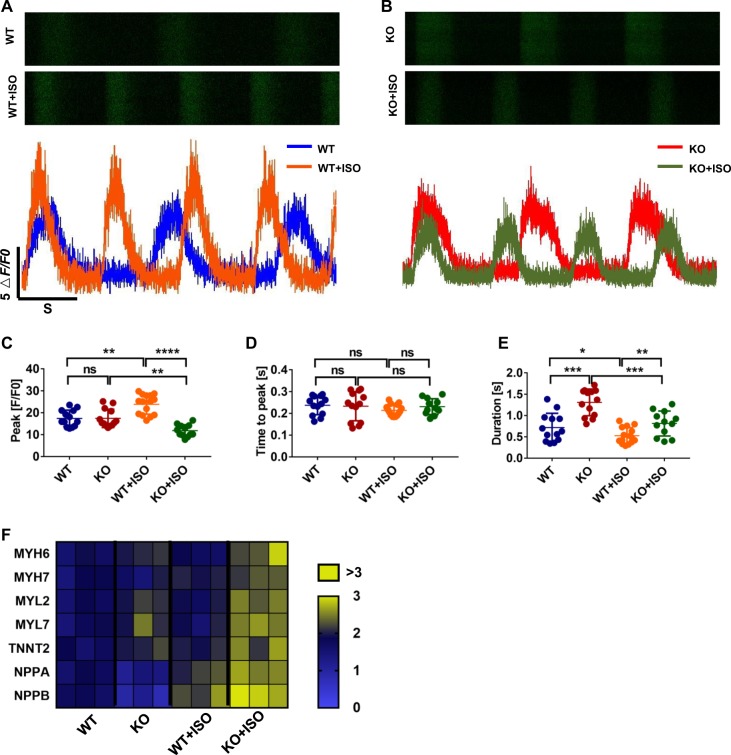
Exacerbation of HF in response to β-adrenergic stimulation in MLP-deficient hESC-CMs. **a**, **b** Representative line-scan images from WT and MLP KO hESC-CMs after 1 week of 10 μM ISO treatment beginning at day 15 of cardiac differentiation. **c**–**e** Quantification of peak, time to peak, and calcium transient duration in WT, MLP KO, WT/ISO, and MLP KO/ISO hESC-CMs (WT *n* = 13, WT/ISO *n* = 15, MLP KO *n* = 13, and MLP KO/ISO *n* = 12). **e** Heatmap representations of hypertrophy related gene expression in WT, MLP KO, WT/ISO, and MLP KO/ISO hESC-CMs (*n* = 3). Results are presented as means ± S.E.M. of three independent experiments. *P < 0.05; **P < 0.01; ***P < 0.001; ****P < 0.0001; ns, not significant, unpaired two-sided Student’s *t* test

Chronic administration of β-adrenergic agonists, such as isoproterenol^37^, have been shown to aggravate HCM and induce HF in HCM models of disease^38^. Deletion of the β2-adrenergic receptor^39^ and overexpression of a β-adrenergic receptor kinase 1 inhibitor^40^ has been shown to prevent the development of cardiomyopathy in MLP-deficient mice. We thus examined whether treatment with ISO could accelerate the development of HCM disease phenotype in MLP KO hESC-CMs. One week of 10 μM ISO treatment from the day 15 of cardiac differentiation resulted in markedly increased calcium release amplitudes of WT hESC-CMs and significantly decreased calcium release amplitudes in MLP KO hESC-CMs (Fig. 6a–c). ISO treatment did not result in significant changes of time to peak in WT and MLP KO hESC-CMs (Fig. 6a, b, d). Although ISO treatment can reduce the duration of calcium transients both in WT and MLP KO hESC-CMs, the duration of calcium transients in MLP KO hESC-CMs was still maintained at higher levels than in WT hESC-CMs (Fig. 6a, b, e). Gene expression of cardiac hypertrophy related genes such as *MYH6, TNNT2, NPPA*, and *NPPB* was modestly induced in ISO-treated WT hESC-CMs, but was found to be markedly elevated in MLP KO hESC-CMs (Figs. 6f and S6A–G). These results suggest that β-adrenergic stimulation can exacerbate HCM disease phenotypes in MLP-deficient hESC-CMs.

### Treatment of Ca^2+^ dysregulation prevents development of the HCM phenotype in MLP-deficient cardiomyocytes

**Fig. 7:**
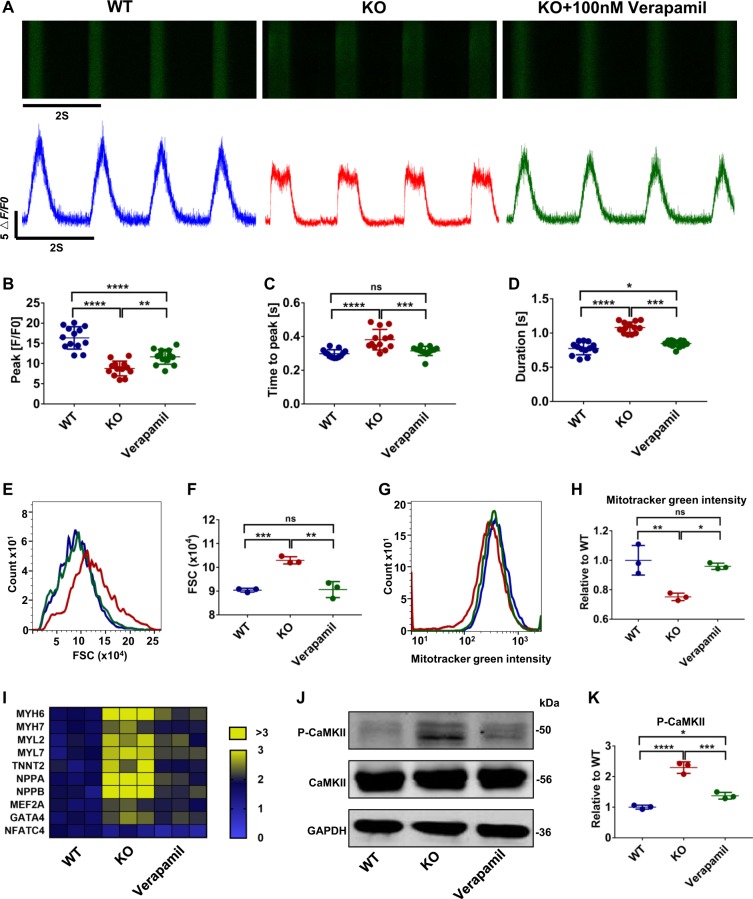
Verapamil rescued HCM phenotype in MLP-deficient CMs. **a** Representative line-scan images in WT (blue), MLP KO (red), and MLP KO hESC-CMs treated with 100 nM verapamil (green) for 10 continuous days beginning at day 20 of cardiac differentiation (*n* = 13). **b**–**d** Quantification of peak, time to peak, and calcium transient duration in WT, MLP KO, and verapamil-treated MLP KO hESC-CMs (*n* = 12). **e**, **f** Forward scatter (FSC) and **g**, **h** Mitotracker green intensity obtained by flow cytometry in WT, MLP KO, and verapamil-treated MLP KO hESC-CMs. **i** Heatmap representations of hypertrophy related gene expression in WT, MLP KO, and verapamil-treated MLP KO hESC-CMs. **j**, **k** Immunoblot analysis of P-CaMKII and quantification of P-CAMKII normalized by GAPDH in WT, MLP KO, and verapamil-treated MLP KO hESC-CMs (*n* = 3). Results are presented as means ± S.E.M. of three independent experiments. *P < 0.05; **P < 0.01; ***P < 0.001; ****P < 0.0001; ns, not significant, unpaired two-sided Student’s *t* test

As Ca^2+^ dysregulation was observed to occur before other hallmarks of HCM disease phenotypes, we next evaluated whether pharmaceutical inhibition of calcium entry with the L-type Ca^2+^ channel blocker verapamil could prevent the development of HCM phenotype in MLP KO hESC-CMs. Continuous addition of verapamil using therapeutic dosages (100 nM) for 10 days beginning at day 20 of cardiac differentiation was found to significantly improve impaired calcium handling function in MLP KO hESC-CMs, including increased rate of calcium uptake and release (Fig. 7a–d). Consistent with higher calcium amplitudes (Fig. 7b), increased SR Ca^2+^ storage capacity was also observed in verapamil-treated MLP KO hESC-CMs as compared with nontreated cells (Fig. S7A–C). Assessment of cell size and mitochondrial content by flow cytometry showed that administration of verapamil ameliorated cell enlargement (Fig. 7e, f) and mitochondrial damage (Fig. 7g, h) in MLP KO hESC-CMs. Treatment with verapamil was also found to downregulate the expression of genes related to cardiac hypertrophy (Figs. 7i and S7D) and reduce activation of the hypertrophic signaling pathways (Fig. 7j, k). These results suggest early intervention to treat calcium handling deficiencies can help prevent the development of HCM disease phenotypes in MLP-deficient hESC-CMs.

## Discussion

In this study, we report the development of an in vitro human model for MLP-associated disease using CRISPR/Cas9 to genetically edit *CSRP3*^−/−^ H9 hESCs and generate MLP KO cardiomyocytes. As prior preclinical models for MLP deficiency have been limited to transgenic animals, our system allows for the analysis of functional changes and molecular mechanisms underlying MLP deficiency in a human cardiac model of disease for the first time. Specifically, we show that MLP-deficient hESC-CMs develop disease phenotypes associated with HCM and that abnormal calcium handling is a central mechanism underlying disease pathogenesis. Treatment with the calcium channel blocker verapamil was found to prevent development of the disease phenotype in MLP-deficient CMs through maintenance of calcium homeostasis.

**Fig. 8:**
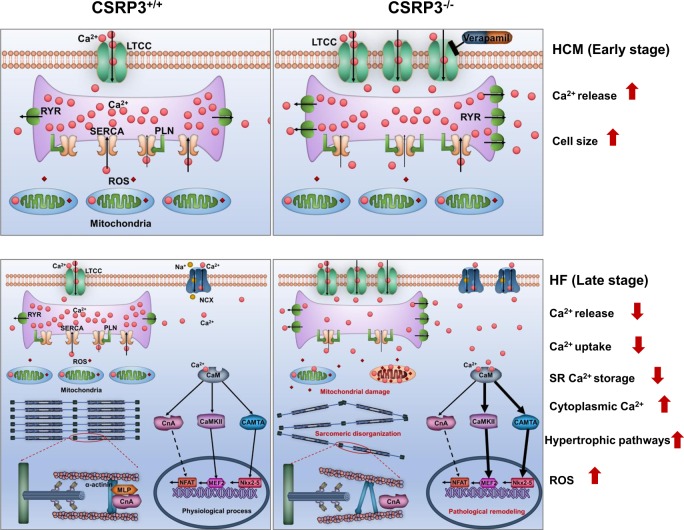
Schematic illustrating the pathogenesis underlying the disease phenotype of MLP-deficiency in MLP KO hESC-CMs. Abnormal calcium handling and elevation of cytoplasmic Ca^2+^ concentrations are the central mechanism for pathogenesis of HCM and HF in MLP KO hESC-CMs. LTCC, L-type calcium channels; NCX, sodium-calcium exchange pump; CaM, Calmodulin; CnA, calcineurin

Given our findings in MLP KO hESC-CMs, we propose a model to illustrate the pathogenesis underlying MLP-associated HCM and HF (Fig. 8). Based on our findings, we suggest that disruption of intracellular Ca^2+^ homeostasis is an initial inciting event. Prior to the onset of HCM phenotypes, transcriptional expression of CACNA1C (encoding the alpha subunit of L-type calcium channel) was significantly elevated in MLP-deficient hESC-CMs, leading to higher levels of extracellular Ca^2+^ entering the cytoplasm during systole, and triggering the SR to release more Ca^2+^. Higher amplitude of calcium transients in MLP-deficient hESC-CMs was subsequently observed as early as day 15 of cardiac differentiation as compared with WT hESC-CMs. Similarly, SERCA expression was observed to be decreased at both the mRNA and protein levels in MLP-deficient hESC-CMs, resulting in reduced cytoplasmic Ca^2+^ transport back into the SR, and resulting in the elevation of diastolic [Ca^2+^]. As a result, SR Ca^2+^ load in MLP-deficient hESC-CMs was observed to be decreased because of increased RyR Ca^2+^ leak and diminished SR uptake. As a consequence of defective SR Ca^2+^ cycling, Ca^2+^ transients in MLP-deficient CMs show distinct alterations such as decreased amplitude, prolonged time to peak, reduced the rate of Ca^2+^ removal, and elevated resting [Ca^2+^], consistent with previously reported findings in failing CMs^41^. Prolonged calcium uptake and increased cytoplasmic calcium concentration in MLP-deficient hESC-CMs obstruct sarcomeric relaxation, leading to diastolic dysfunction.

Several Ca^2+^-dependent pathways including calmodulin, CaMKII, and calcineurin have been shown to be key regulators of cardiomyopathy^23^. Cytoplasmic Ca^2+^ binds to calmodulin, which induces expression of ANF by calmodulin-binding transcriptional activator 2 (CAMTA2). In addition, CAMTA2 participates in hypertrophic response by binding to NKX2-5^42^. Ca^2+^ binding to calmodulin also activates Ca^2+^-sensitive enzymes such as calcineurin and CaMKII, which have been shown to be overactivated in HCM and HF^27,28^. In line with these findings, MLP-deficient hESC-CMs were observed to have increased expression of calmodulin and phosphorylated CAMKII. In our cell models, overactivation of Ca^2+^-Calmodulin-CaMKII signaling in MLP-deficient CMs is most likely due to elevated cytoplasmic Ca^2+^. However, we found calcineurin was not increased at the protein level in MLP-deficient hESC-CMs. A possible explanation is that MLP, calcineurin, and calsarcin-1 can form a trimeric complex at the Z-disk and MLP is essential for calcineurin anchorage to the Z-disk^43^. Compartmentalization of calcineurin at the Z-disk is essential for coordinated dephosphorylation of NFAT transcription factors, which also are localized at the Z-disk^44,45^. Calcineurin was observed to be dislocated from the Z-disk in MLP-deficient hESC-CMs, which could inhibit the activation of Ca^2+^-calcineurin–NFAT signaling.

Resting Ca^2+^ can also be transported into the mitochondria by mitochondrial Ca^2+^ uniporters^46^. Elevated resting [Ca^2+^] in MLP-deficient CMs leads to even higher Ca^2+^ transport into the mitochondria, which has been shown to damage mitochondria^34^. MLP-deficient hESC-CMs demonstrate hallmarks of mitochondrial injury such as decreased mtDNA/nDNA ratio, Mitotracker green intensity, and reduced mitochondria density. Mitochondrial fragmentation was observed in MLP-deficient hESC-CMs, mimicking findings observed in patient HF^47^. Increased ROS were also observed in MLP-deficient hESC-CMs. Elevated mitochondrial ROS levels and ROS mediated damage has been demonstrated both in patients with HF and in preclinical models of the disease^48,49^. Increased ROS in MLP-deficient CMs can be explained by the several hypotheses. Frist, abnormal mitochondria observed in MLP-deficient CMs are a major source of ROS production. Second, increased energy demand due to inefficient sarcomeric ATP utilization in HCM^31^ require higher output of mitochondria, which could lead to further generation of ROS. It is known that Ca^2+^ uptake by SERCA, NCX, and Ca^2+^ ATPases in diastole are a process requiring significant energy consumption^50^. However, less ATP production due to reduction in mitochondrial density and injury in MLP-deficient hESC-CMs could hinder this process causing a further increase in cytoplasmic Ca^2+^, further forming a positive-feedback loop and thereby aggravating the progression of HCM and HF. Reduced ATP production and increased energy consumption in MLP-deficient CMs result in an increased AMP/ATP ratio, which can activate AMPK as evidenced by increased levels of phosphorylated AMPK.

Genetically modified PSC-derived CMs circumvent the limitations associated with transgenic animal models of human heart disease and have been used to model numerous genetic cardiovascular disorders in vitro, including HCM^21^ and long QT syndrome^37^. However, genetically modified PSC models are associated with several challenges in accurately modeling human heart disease. First, hESC-CMs are developmentally immature, and are characterized by transcriptional profiles more similar to fetal human CMs than adult CMs^13^. Second, hESC-CMs are not at the stage where they can model disease phenotypes at the tissue and organ levels such as interstitial fibrosis and endothelial cell dysfunction. Lastly, mutations in over 100 causal genes have been identified to engender cardiomyopathy in human patients, and our findings may only be specific to *CSRP3* defects resulting in MLP deficiency. In spite of these limitations, our findings are the first to provide evidence of elevated cytoplasmic [Ca^2+^] as an initiating factor in the development of HCM and HF phenotypes caused by MLP deficiency in human CMs. These findings further demonstrate a possible mechanism to prevent development of this disease by early intervention via normalization of Ca^2+^ dysfunction.

## Materials and methods

### Cell culture and cardiac differentiation

H9 and derivative lines were maintained on feeder-free Matrigel (Corning, USA) and fed daily with E8 medium (Cellapy, China). Cells were routinely passaged every 3 days at 70–80% confluency using 0.5 mM EDTA in PBS without MgCl_2_ or CaCl_2_ (HyClone, USA). The cells were cultured at 37 °C with 5% CO_2_. hESCs were differentiated into ESC-CMs using a small molecule-based method as previously described^13^. hESC-CMs were purified with the lactate metabolic-selection method^51^.

### Genome editing

Single-guide RNAs targeting *CSRP3* were designed using online tools (http://crispr.mit.edu). Two sgRNAs (TGGGGCGGAGGCGCAAAATG and ATGGTAGACGGTCTTTTCAC) were tested for editing efficiency, and the first one was selected for genome editing in H9 cells. sgRNA was cloned into the epiCRISPR plasmid as previously reported^11^. Briefly, 2 × 10^6^ H9 cells were disassociated using 0.5 mmol/L EDTA, then these cells were electroporated with 2.5 μg epiCRISPR plasmid in 82 μL stem cell P3 solution + 18 μL supplement (Lonza, Germany) using 4D nucleofector system with the CA137 program (Lonza). Transfected cells were seeded onto Matrigel-coated 10 cm petri dish and cultured in E8 medium with 10 µM Rho kinase inhibitor Y-27632 for the first 24 h. At day 3, cells were selected by puromycin (0.3 μg/mL). The surviving colonies were picked into a 24-well plate and expanded for PCR screening.

Generation of H9-GCaMP and *CSRP3*^−/−^-GCaMP: 2.5 μg AAVS1_sgRNA plasmid (Addgene, #100554, USA) and 2.5 μg pAAVS1-PC-GCaMP6f plasmid (Addgene, #73503) were electroporated together into H9 and CSRP3^−/−^ ESCs. Methods for puromycin screening, selection, and identification of clones are as described above.

### Immunostaining and imaging analyses

The ESC cells and ESC-CMs were fixed with 4% PFA, permeabilized in PBS containing 0.5% Triton X-100 (Sigma, USA) for 15 min, and blocked with 3% BSA (Sigma) for 60 min at room temperature. After that cells were incubated with primary antibodies overnight at 4 °C. Cells were then washed three times using PBS for 5 min each. Then cells were incubated with secondary antibodies diluted in 3% BSA for 1 h at room temperature. After three washes with PBS for 5 min each, cells were counterstained with DAPI (300 nM, Invitrogen, USA) for 5 min. Images were taken under Leica DMI 4000B. The full list of primary and secondary antibodies used and their appropriate dilution are provided in Table S2.

### Flow cytometry

ESC-CMs were singularized with CardioEasy CM dissociation buffer (Cellapy). Cells were washed three times with PBS and fixed with chilled (4 °C) fixation buffer (BD Biosciences) for 10 min at room temperature. Next, the samples were incubated with the primary antibody (cTnT) for 30 min. After three washes with PBS, cells were incubated with the secondary antibody (Alexa Fluor 488) for 30 min. Then the samples were washed in PBS and assessed using FACS analysis (EPICS XL, Beckman).

Quantification of mitochondria and reactive oxygen species (ROS): Dissociated hESC-CMs were stained using 50 nM Mitotracker green (Beyotime, C1048, China), 10 μM Cell ROS green (Beyotime, S0033), and 2.5 μM MitoSOX Red (Invitrogen, #M36008). Live cells were incubated with these dyes (made in RPMI 1640, Corning) for 20 min at 37 °C and 5% CO_2_. After washed by PBS, cells were resuspended after centrifugation in PBS. Samples were stored on ice until flow cytometry was run on. The results were analyzed with FlowJo X program. Mean signal fluorescence intensity was used to compare mitochondrial content and ROS production in MLP KO CMs relative to WT CMs.

### Ca^2+^ imaging

H9-GCaMP and *CSRP3*^−/−^-GCaMP derived cardiomyocytes were seeded onto confocal dishes. Intracellular calcium flux was imaged using a confocal microscope (Leica, TCS5 SP5, Germany) at 40×. Spontaneous Ca^2+^ transients were acquired at 37 °C and 5% CO_2_ using line-scan mode at a sampling rate of 1 ms/line. A total of 8192 lines were acquired for 8.192 s recoding. For detection of caffeine-evoked Ca^2+^ release, 10 mM caffeine in Ca^2+^ free solution was used to evoke sarcoplasmic reticulum (SR) Ca^2+^ transients in hESC-GCaMP-CMs. The results were analyzed with Image J and Igor.

### RNA extraction and quantitative real-time PCR (qRT-PCR)

Total RNA was extracted from about 2 × 10^5^ cells with TRIzol (Life Technologies, USA) and treated with DNase I (Life Technologies) for about 30 min at 37 °C to eliminate DNA contamination. For qRT-PCR, 1 μg of RNA was reverse transcribed into cDNAs using the PrimeScript^TM^ reverse transcription system (Takara, Japan), following the manufacturer’s instructions. QRT-PCR was performed on iCycler iQ5 (Bio-Rad) using 2× SYBR Master Mix (Takara). The relative quantification of gene expression was carried out according to the ▵CT method. Primer sequences are listed in Table S1.

### Western blots

Cell pellets were washed with cold PBS and resuspended in Mammalian Protein Extraction Reagent (Thermo, #78501, USA) with 5 mM EDTA (Thermo, #1861275), containing a protease inhibitor cocktail (Thermo, #1861278) and phosphatase inhibitor cocktail (Thermo, #1862495). Samples were incubated on ice for 30 min and vibrated every 10 min and then centrifuged at 12,000 rpm for 15 min. The protein concentration of the supernatant was measured by BCA method. Equal amount of denatured protein were resolved by electrophoresis 8–12% (depending on protein size) sodium dodecyl sulfate polyacrylamide gels. The gel was transferred onto a PVDF membrane at 300 mA for 90 min using gel transfer device (Bio-Rad). Then the membrane was blocked with 5% nonfat milk powder at room temperature for 1 h. Membranes were incubated with primary and secondary antibodies of appropriate concentrations listed in Table S2. All primary antibodies were incubated at 4 °C overnight, while secondary antibodies were incubated at room temperature for 1 h.

### Data analysis and statistics

All data are presented as means ± standard errors of the means (S.E.M.). Statistical significance was evaluated using two-sided Student’s *t* test for two groups. Significant differences were considered when *P* < 0.05.

## Supplementary information

Supplemental Figures

Table S1

Table S2

Supplementary figure legends.
