# Supplementary figures and images for: MLP-deficient human pluripotent stem cell derived cardiomyocytes develop hypertrophic cardiomyopathy and heart failure phenotypes due to abnormal calcium handling
Source: Cell Death Dis. 2019 Aug 13;10(8):610. doi: 10.1038/s41419-019-1826-4 (PMC6690906; doi:10.1038/s41419-019-1826-4)

Figure S1

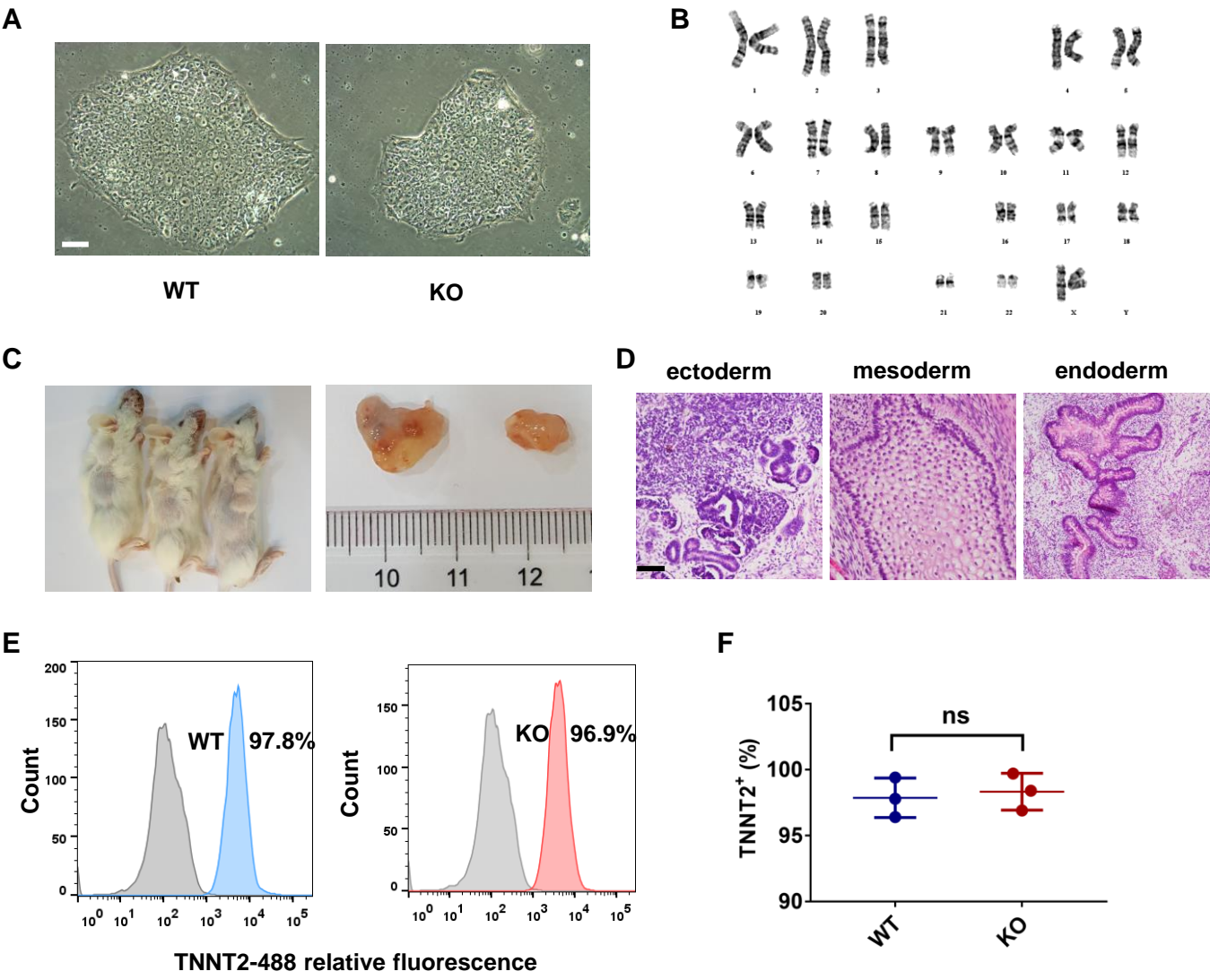

Figure S2

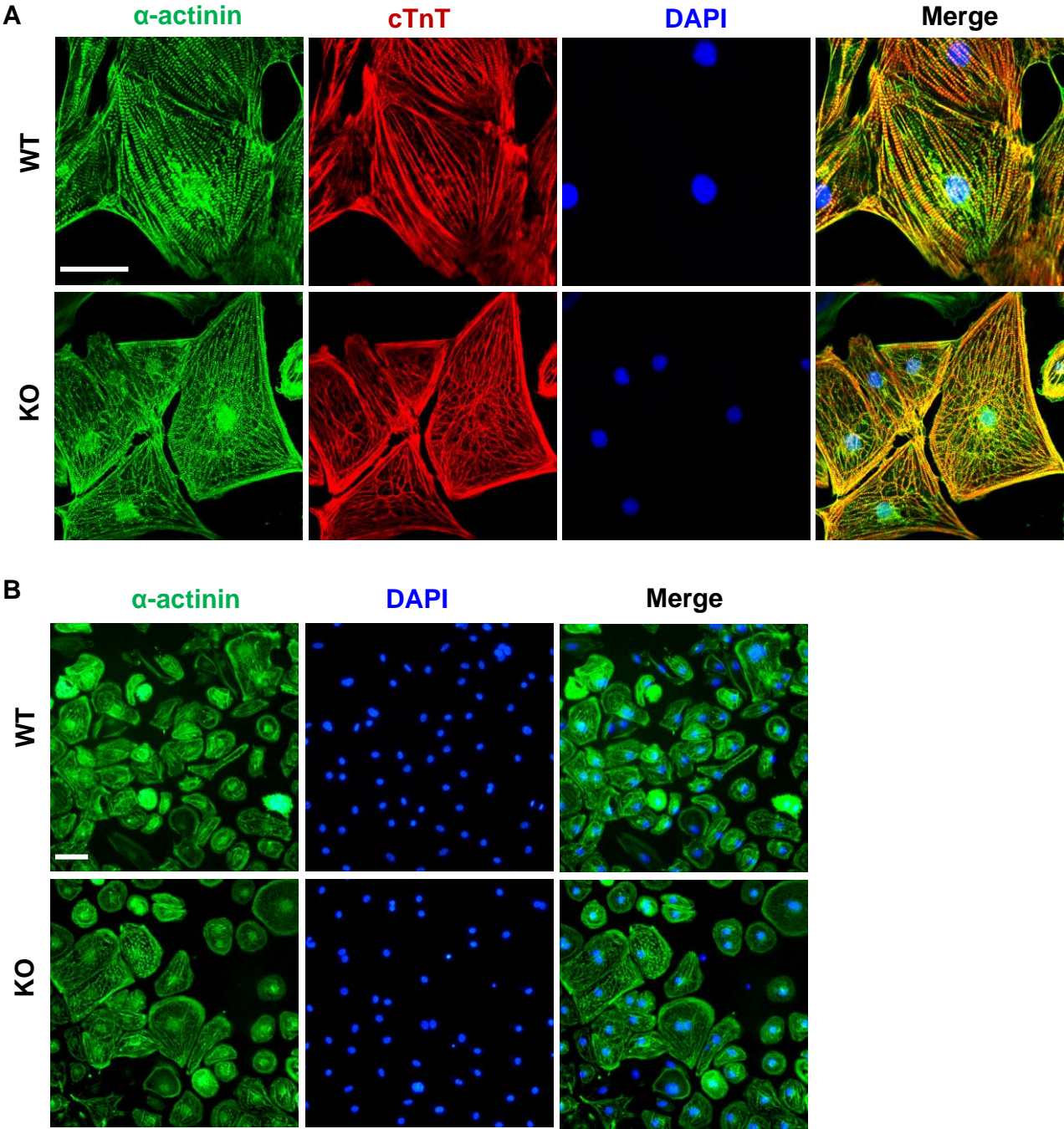

Figure S3

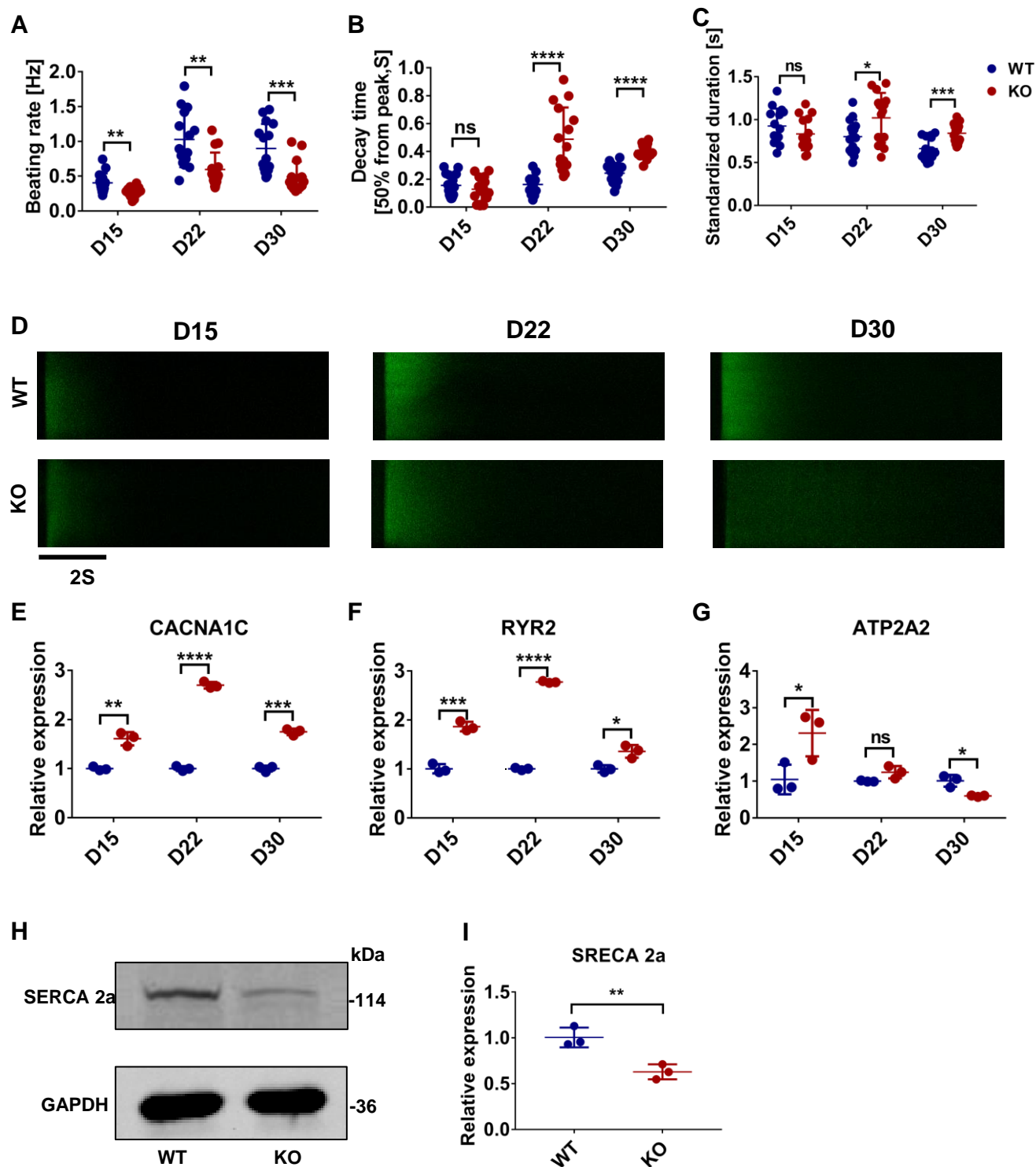

Figure S4

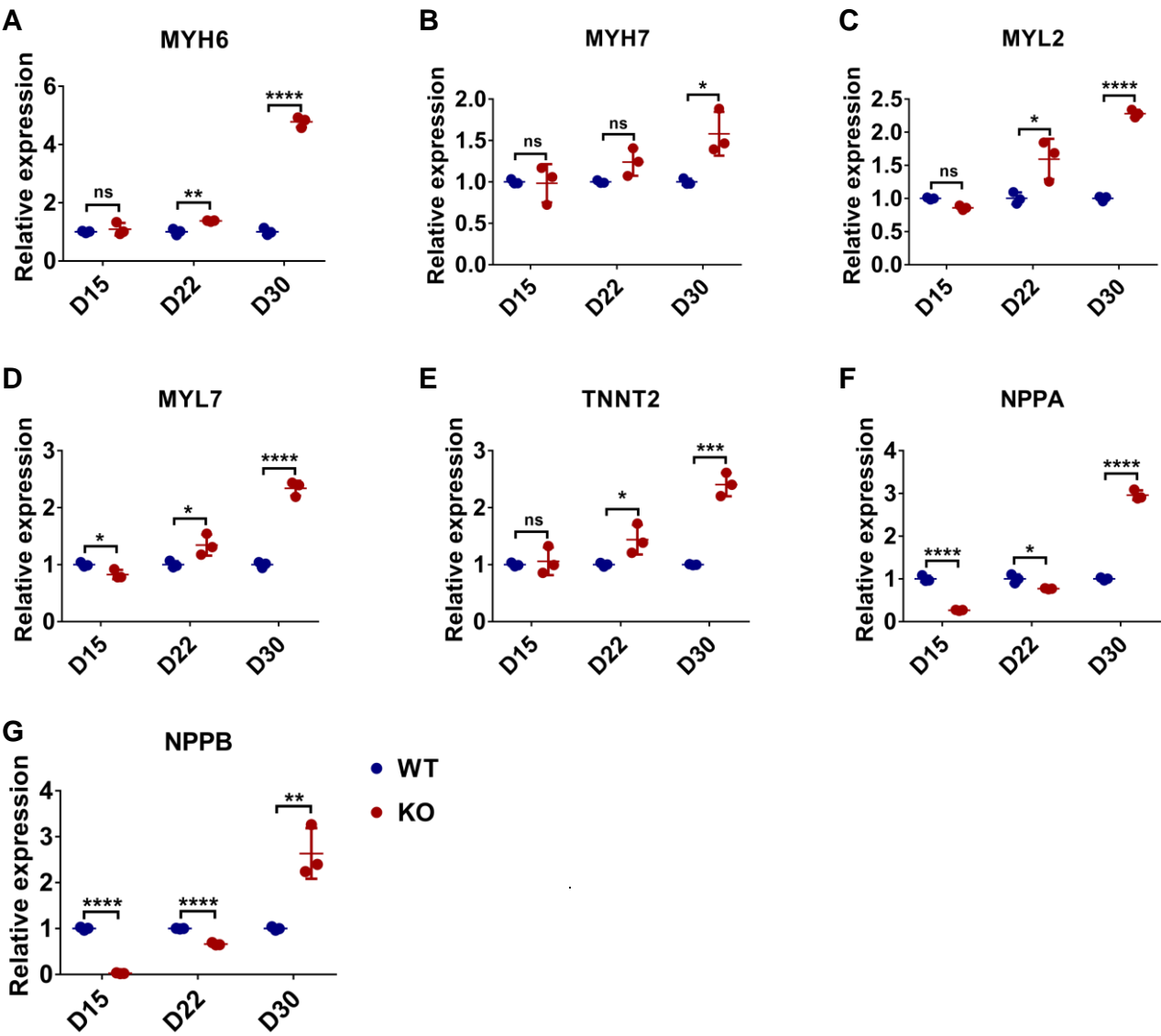

Figure S5

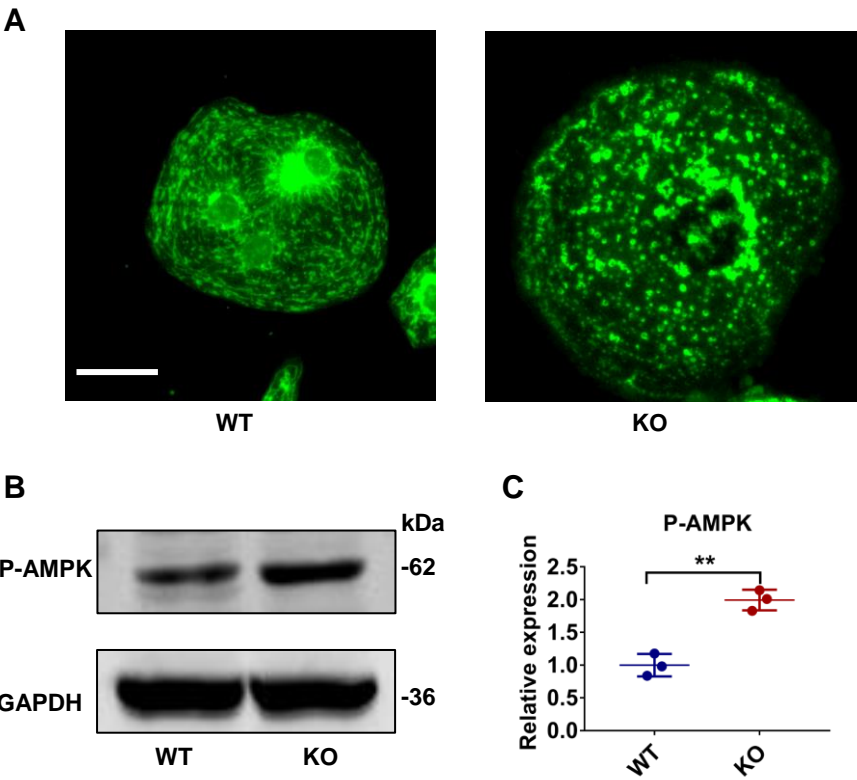

Figure S6

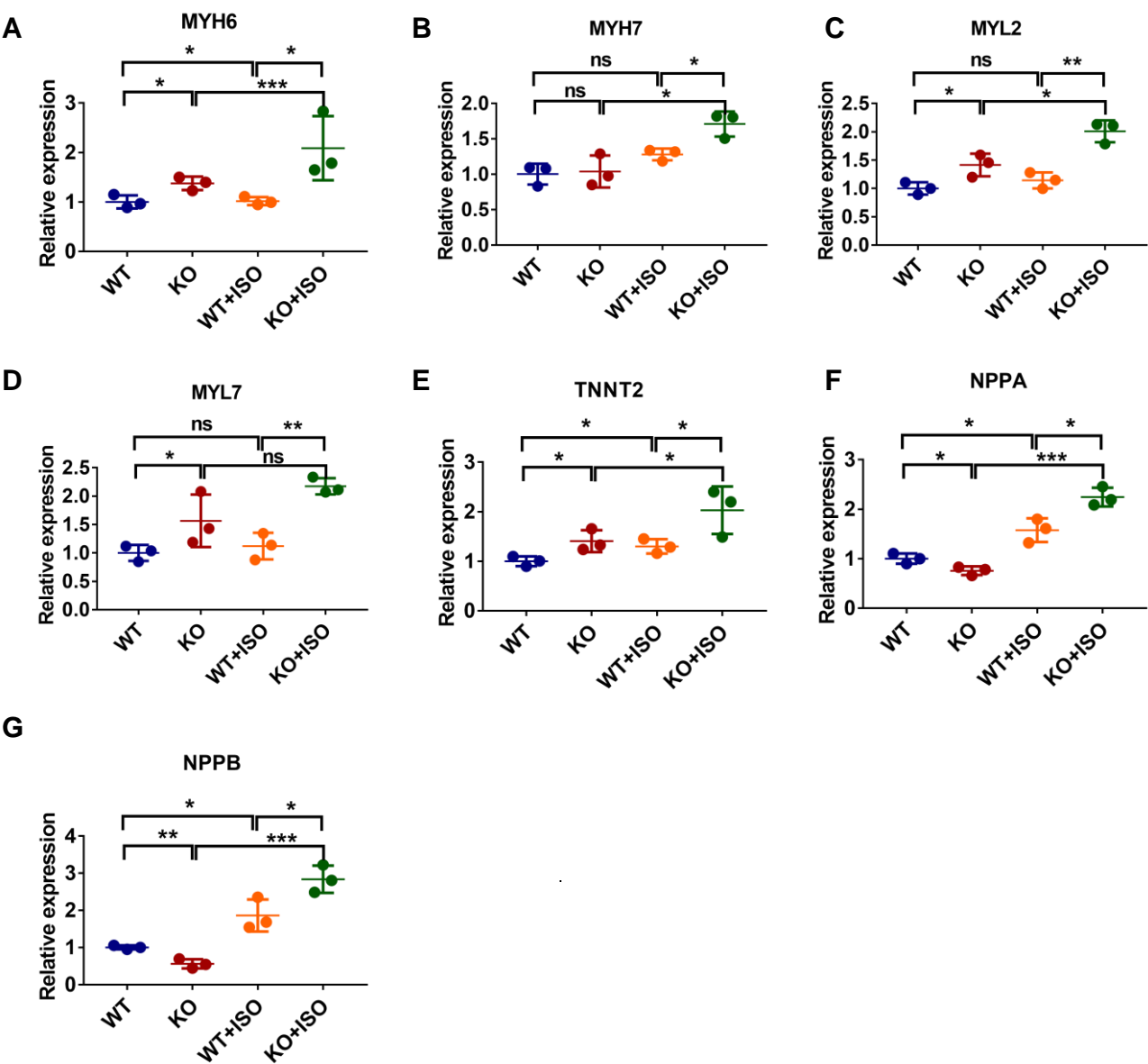

Figure S7

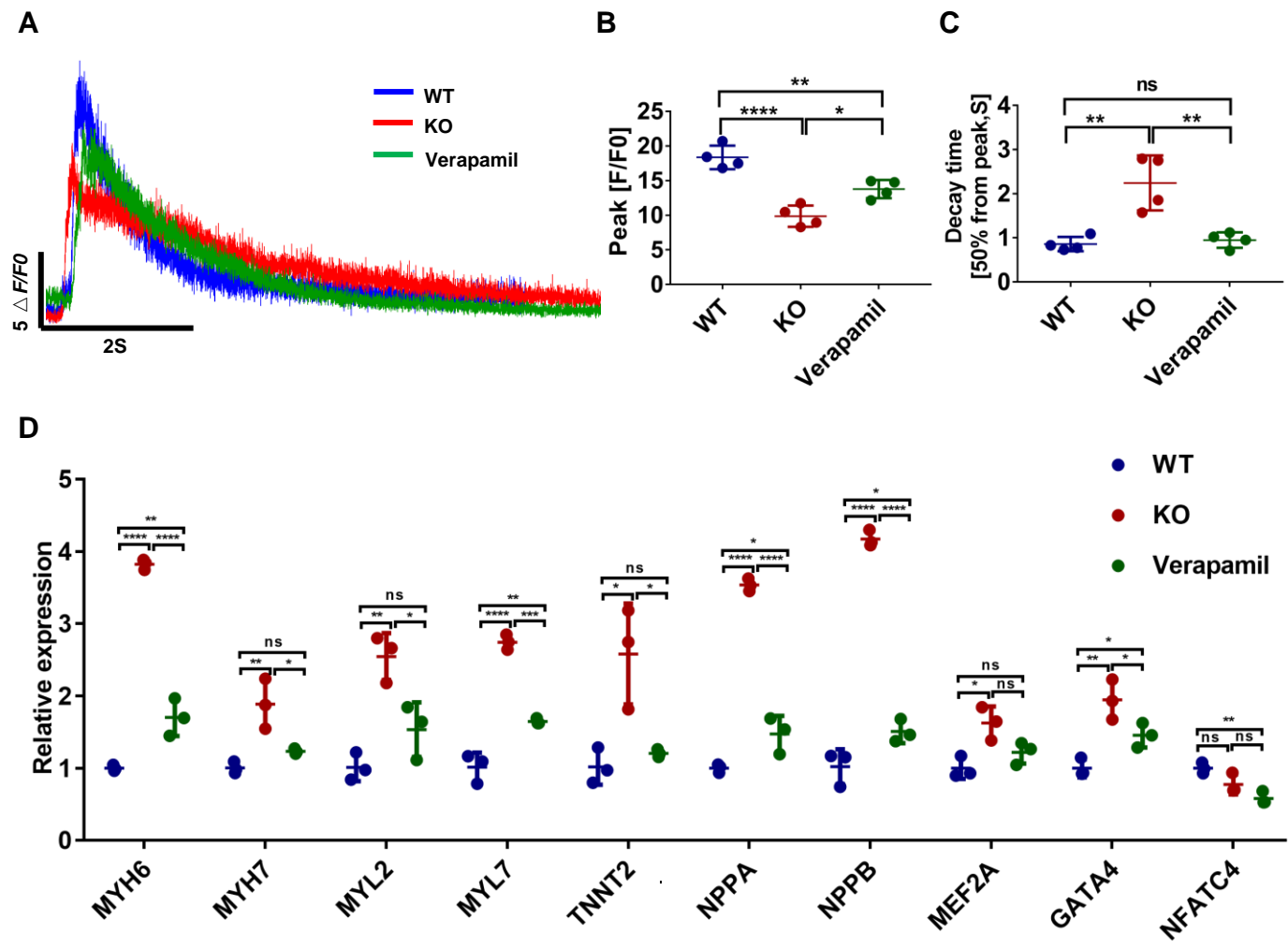

Supplement: Supplementary file 1 — Supplemental Figures [file 41419_2019_1826_MOESM1_ESM.pdf]
