# Supplementary material for: MLP-deficient human pluripotent stem cell derived cardiomyocytes develop hypertrophic cardiomyopathy and heart failure phenotypes due to abnormal calcium handling
Source: Cell Death Dis. 2019 Aug 13;10(8):610. doi: 10.1038/s41419-019-1826-4 (PMC6690906; doi:10.1038/s41419-019-1826-4)
Supplement: Supplementary file 2 — Table S1 [file 41419_2019_1826_MOESM2_ESM.pdf]

Table S1. Primer sequences used for q-PCR

Gene	Forward 5'-3'	Reverse 3'-5'
ND1	ATGGCCAACCTCCTACTCCTCATT	TTATGGCGTCAGCGAAGGGTTGTA
ND2	CCATCTTTGCAGGCACACTCATCA	ATTATGGATGCGGTTGCTTGCGTG
ACTB	CATGTACGTTGCTATCCAGGC	CTCCTTAATGTCACGCACGAT
MYH6	TCTCCGACAACGCCTATCAGTAC	GTCACCTATGGCTGCAATGCT
MYH7	GGCAAGACAGTGACCGTGAAG	CGTAGCGATCCTTGAGGTTGTA
ACTA2	CTATGCCTCTGGACGCACAACT	CAGATCCAGACGCATGATGGCA
NPPA	ACAATGCCGTGTCCAACGCAGA	CTTCATTCGGCTCACTGAGCAC
NPPB	TCTGGCTGCTTTGGGAGGAAGA	CCTTGTGGAATCAGAAGCAGGTG
MYL2	TACGTTCGGGAAATGCTGAC	TTCTCCGTGGGTGATGATG
MYL7	CCGTCTTCCTCACGCTCTT	TGAACTCATCCTTGTTCACCAC
TNNT2	TTCACCAAAGATCTGCTCCTCGCT	TTATTACTGGTGTGGAGTGGGTGTGG
ACTA1	AGGTCATCACCATCGGCAACGA	GCTGTTGTAGGTGGTCTCGTGA
GATA4	GCGGTGCTTCCAGCAACTCCA	GACATCGCACTGACTGAGAACG
MEF2A	CAAGGGCATGATGCCTCCACTA	GCTGAGTACACAAGTCCTTGCG
MEF2C	TCCACCAGGCAGCAAGAATACG	GGAGTTGCTACGGAAACCACTG
ATP2B4	CTCACCGAACTGACCTGTATCG	GGCTGTGTTGATGTTGTCACCTG
RYR2	AGAACTTACACACGCGACCTG	CATCTCTAACCGGACCATACTGC
ATP2A2	GATCACACCGCTGAATCTG	AGTATTGCGGGTTGTTCCAG
CACNA1C	CAGAGGCTACGATTTGAGGA	GCTTCACAAAGAGGTCGTGT
PLN	AGCACGTCAAAAGCTACAGAATCT	CTGATGTGGCAAGCTGCAGATC
NCX1	TCGAGATTGTCTCTTCGGGC	CATATGCAAACACCGAGGCG
CAMK2D	ACACGGTGACTCCTGAAGCCAA	GTCTCCTGTCTGTGCATCATGG
PPP3CA	GCCCTGATGAACCAACAGTTCC	GCAGGTGGTTCTTTGAATCGGTC
CALM1	CCAACAGAAGCTGAATTGCAGGA	CAAAGACTCGGAATGCCTCACG
MCIP1	GGAAACAAGTGGAAGATGCGACC	TGTCAGTCGCTGCGTGCAATTC
NFATC4	GCACCGTATCACAGGCAAGATG	TCAGGATTCCCGCGCAGTCAAT
COL1A1	GATTCCCTGGACCTAAAGGTGC	AGCCTCTCCATCTTTGCCAGCA
COL4A1	TGTTGACGGCTTACCTGGAGAC	GGTAGACCAACTCCAGGCTCTC
CUL1	CAATGACGCTGGCTTTGTGGCT	CAAGGAGTCACAGTATCGAGCC
BECN1	CTGGACACTCAGCTCAACGTCA	CTCTAGTGCCAGCTCCTTTAGC
CHMP2B	GGCTATAATCAGAGATCGAGCAG	CTCGTCTTCTGTTTCCGTAGATG
LAMP1	CGTGTCACGAAGGCGTTTTCAG	CTGTTCTCGTCCAGCAGACACT
LAMP2	GGCAATGATACTTGTCTGCTGGC	GTAGAGCAGTGTGAGAACGGCA

SKP1	TCCTGAGGAGATTCGCAAGACC	ACTTCTCTTCACACCACTGGTTC
FBXO32	CACTGGTCCAAAGAGTCGGCAA	GCACAAAGGCAGGTCAGTGAAG
SCN5A	CTGACCTCACCATCACTATGTG	GCTGTGAAAATCCCTGTGAAG
KCNH2	AATCGCCTTCTACCGGAAAG	CACCATGTCCTTCTCCATCAC
KCNQ1	TCTGTCTTTGCCATCTCCTTC	CCTCCATGCGGTCTGAATG
KCNJ2	AAGACGGTATGAAGTTGGCC	CGGGTGTGGACTTTACTCTTC
KCNA5	GTTCCGCATCTTCAAGCTCTCC	CGAAGTAGACGGCACTGGAGAA
CX43	GGAGATGAGCAGTCTGCCTTTC	TGAGCCAGGTACAAGAGTGTGG
GAPDH	GGAGCGAGATCCCTCCAAAAT	GGCTGTTGTCATACTTCTCATGG
