# Supplementary material for: MLP-deficient human pluripotent stem cell derived cardiomyocytes develop hypertrophic cardiomyopathy and heart failure phenotypes due to abnormal calcium handling
Source: Cell Death Dis. 2019 Aug 13;10(8):610. doi: 10.1038/s41419-019-1826-4 (PMC6690906; doi:10.1038/s41419-019-1826-4)
Supplement: Supplementary file 3 — Table S2 [file 41419_2019_1826_MOESM3_ESM.pdf]

Table S2. Primary and Secondary Antibodies

Type	Antibody	Application	Dilution	Species	Manufacturer And Catalog Number
Primary	Anti-OCT4	Immunofluorescence	1:100	Rabbit Polyclonal	Santa Cruz sc-9081
	Anti-TRA-1-81	Immunofluorescence	1:100	Mouse Monoclonal	Santa Cruz sc-21706
	Anti-MLC2v	Immunofluorescence	1:50	Rabbit Polyclonal	Proteintech 10906-1-AP
	Anti-*CSRP3*	Western blot	1:1000	Mouse Monoclonal	Santa Cruz sc-166930
	Anti-MLC2a	Immunofluorescence	1:100	Mouse Monoclonal	Santa Cruz sc-365255
	Anti-α-actinin	Immunofluorescence	1:100	Rabbit Polyclonal	Abcam Ab137346
		Western blot	1:1000		
	Anti-cTnT	Immunofluorescence	1:100	Mouse Monoclonal	Abcam Ab8295
		Western blot	1:1000		
		Flow cytometry	1:200		
	Anti-SERCA2	Western blot	1:1000	Rabbit Monoclonal	Cell signaling #9580
	Anti-AMPK	Western blot	1:1000	Rabbit Polyclonal	Abcam Ab39400
	Anti-Calcineurin	Western blot	1:1000	Rabbit Polyclonal	Cell signaling #2614
	Anti-Phospho -CaMKII	Western blot	1:1000	Rabbit Monoclonal	Cell signaling #12716
	Anti-CaMKII	Western blot	1:1000	Rabbit Monoclonal	Abcam Ab52476
	Anti-MYH7	Western blot	1:1000	Mouse Monoclonal	Abcam Ab 174640
	Anti-GAPDH	Western blot	1:1000	Mouse Monoclonal	Santa Cruz sc-365062
	Goat anti-Mouse IgG Alexa Fluor 594	Immunofluorescence	1:200	Goat anti-Mouse IgG	Invitrogen A21145
	Goat anti-Rabbit IgG Alexa Fluor 488	Immunofluorescence	1:200	Goat anti-Rabbit IgG	Invitrogen A32731
	Chicken anti-Rabbit IgG	Immunofluorescence	1:200	Chicken anti-Rabbit IgG	Invitrogen A21442

Secondary	Alexa Fluor 594				
	Chicken anti-Mouse IgG Alexa Fluor 488	Immunofluorescence	1:200	Chicken anti-Mouse IgG	Invitrogen A21200
	Goat anti-Rabbit IgG (H + L) IRDye 800CW	Western blot	1:20000	Goat anti-Rabbit IgG	LI-COR 926-32211
	Goat anti-Mouse IgG (H + L) IRDye 800CW	Western blot	1:20000	Goat anti-Mouse IgG	LI-COR 926-32210
