# Supplementary material for: MLP-deficient human pluripotent stem cell derived cardiomyocytes develop hypertrophic cardiomyopathy and heart failure phenotypes due to abnormal calcium handling
Source: Cell Death Dis. 2019 Aug 13;10(8):610. doi: 10.1038/s41419-019-1826-4 (PMC6690906; doi:10.1038/s41419-019-1826-4)
Supplement: Supplementary file 4 — Supplementary figure legends. [file 41419_2019_1826_MOESM4_ESM.docx]

**Supplementary Figure 1. Characterization of MLP KO hESCs and hESC-CM differentiation efficiency.**

(A) Representative images of WT and MLP KO hESC colonies. Scale bar, 100 μm. (B) 46 X, X normal karyotype of CSRP3 MLP KO hESCs. (C) CSRP3 MLP KO hESCs injected under the skin of immunodeficient mice form teratomas. (D) Teratomas contain cell lineages from three germ layers (ectoderm, mesoderm and endoderm). Scale bars, 100μm. (E, F) Flow cytometric staining for TNNT2 in representative WT and MLP KO hESC-CMs after purification at day 15 of cardiac differentaiton. Results are presented as means ± S.E.M. of 3 independent experiments. ns, not significant, unpaired two-sided Student`s t-test.

**Supplementary Figure 2. MLP-deficient hESC-CMs recapitulate HCM disease phenotypes *in vitro*.**

(A) Immunostaining of sarcomeric α-actinin (green) and cTnT (red) show a higher proportion of disorganized myofilament ultrastructure in MLP KO hESC-CMs at day 30 of cardiac differentiation. Scale bars, 50μm. (B) Immunostaining for α-actinin shows an increased cellular size in MLP KO hESC-CMs at day 30. Scale bars, 50μm.

**Supplementary Figure 3. MLP-deficient hESC-CMs exhibit abnormal Ca2+ handling properties and decreased SERCA 2a expression.**

(A-C) Beating rate, decay time and calcium transient duration standardized by beating rate in WT-GCaMP and MLP KO-GCaMP hESC-CMs at day 15, day 22, and day 30 of cardiac differentiation (n=15 cells per group). Standardized duration = duration/(60/HR)^0.5^. (D) Representative line-scan images induced with 10 mM caffeine in WT-GCaMP and MLP KO-GCaMP hESC-CMs at day 15, day 22, and day 30 of cardiac differentiation. (E-G) q-PCR analysis of CACNA1C, RYR2 and ATP2A2 in WT and MLP KO CMs at day 15, day 22 and day 30. (H) Protein blot analysis and (I) quantification of SERCA 2a in WT and MLP KO hESC-CMs at day 30. Results are presented as means ± S.E.M. of 3 independent experiments. *P < 0.05; **P < 0.01; ***P < 0.001; ****P < 0.0001; ns, not significant, unpaired two-sided Student`s t-test.

**Supplementary Figure 4. Transcriptional analysis of HCM related genes.**

(A-G) q-PCR analysis of hypertrophy-related genes in WT and MLP KO hESC-CMs at day 15, day 22 and day 30 of cardiac differentiation. Results are presented as means ± S.E.M. of 3 independent experiments. *P < 0.05; **P < 0.01; ***P < 0.001; ****P < 0.0001; ns, not significant, unpaired two-sided Student`s t-test.

**Supplementary Figure 5. MLP-deficient hESC-CMs exhibit mitochondrial damage and energy depletion.**

(A) Mitotracker green images showed punctate and fragmented mitochondria in MLP KO hESC-CMs at day 30 of cardiac differentiation. Scale bars, 50μm. (B) Protein blot analysis of phosphorylated AMPK in WT and MLP KO hESC-CMs at day 30. (C) Quantification of P-AMPK normalized by GAPDH from 3 independent experiments showed higher phosphorylated AMPK level in MLP KO hESC-CMs. Results are presented as means ± S.E.M. of 3 independent experiments. **P < 0.01, unpaired two-sided Student`s t-test.

**Supplementary Figure 6. Transcriptional analysis of HCM related genes in** **WT, MLP KO, WT/ISO and MLP KO/ISO hESC-CMs.**

(A-G) q-PCR analysis of hypertrophy-related genes in WT, MLP KO, WT/ISO and MLP KO/ISO hESC-CMs. Results are presented as means ± S.E.M. of 3 independent experiments. *P < 0.05; **P < 0.01; ***P < 0.001; ns, not significant, unpaired two-sided Student`s t-test.

**Supplementary Figure 7. Verapamil prevents development of the HCM phenotypes in MLP deficient hESC-CMs.**

(A) Representative Ca^2+^ transients induced with 10 mM caffeine and (B, C) quantification of peak amplitude and decay time of caffeine-evoked Ca^2+^ transients in WT-GCaMP, MLP KO-GCaMP and verapamil treated MLP KO hESC-CMs (n=4). (D) q-PCR analysis of HCM related genes in WT, MLP KO and verapamil treated MLP KO hESC-CMs at day 30 of cardiac differentiation. Results are presented as means ± S.E.M. of 3 independent experiments. *P < 0.05; **P < 0.01; ***P < 0.001; ****P < 0.0001; ns, not significant, unpaired two-sided Student`s t-test.
